# Assessment System for Child Head Injury from Falls Based on Neural Network Learning

**DOI:** 10.3390/s23187896

**Published:** 2023-09-15

**Authors:** Ziqian Yang, Baiyu Tsui, Zhihui Wu

**Affiliations:** 1 College of Furnishings and Industrial Design, Nanjing Forestry University, Nanjing 210037, China; 2 Jiangsu Co-Innovation Center of Efficient Processing and Utilization of Forest Resources, Nanjing 210037, China

**Keywords:** head injury from falls, deep learning, Open Pose, LSTM, 3D transform model

## Abstract

Toddlers face serious health hazards if they fall from relatively high places at home during everyday activities and are not swiftly rescued. Still, few effective, precise, and exhaustive solutions exist for such a task. This research aims to create a real-time assessment system for head injury from falls. Two phases are involved in processing the framework: In phase I, the data of joints is obtained by processing surveillance video with Open Pose. The long short-term memory (LSTM) network and 3D transform model are then used to integrate key spots’ frame space and time information. In phase II, the head acceleration is derived and inserted into the HIC value calculation, and a classification model is developed to assess the injury. We collected 200 RGB-captured daily films of 13- to 30-month-old toddlers playing near furniture edges, guardrails, and upside-down falls. Five hundred video clips extracted from these are divided in an 8:2 ratio into a training and validation set. We prepared an additional collection of 300 video clips (test set) of toddlers’ daily falling at home from their parents to evaluate the framework’s performance. The experimental findings revealed a classification accuracy of 96.67%. The feasibility of a real-time AI technique for assessing head injuries in falls through monitoring was proven.

## 1. Introduction

For toddlers three years and younger, falls pose the most significant risk to their lives, leading to injuries such as craniofacial fractures, concussions, and sprains of the cervical spine’s spinal cord and neck strains [1,2,3,4,5]. According to an Injury Review of Chinese Children and Adolescents [6], falls are the most common reason for accidental injury and death in China’s children aged 1 to 4 years old. A total of 40.18 percent of these falls occurred at home, and 46.99 percent resulted in head injuries. Toddlers are most likely to fall from steps, daycare furniture, or playground equipment occasionally during normal development [7]. Among them, toddlers climbing over bed guardrails, tumbling, and sustaining injuries represent a high incidence of events nationwide. The majority of toddler overturned falls involve head contact.

Research on fall injury detection can be categorized into the wearable sensor-based method (WSM), the finite element method (FEM), and visual detection. In wearable sensor-based systems, accelerometers and gyroscopes are commonly employed to measure acceleration and deflection angles. Gina et al. [8] recorded incidences of falling in children aged 12 to 36 months who wore sensor-laden headbands (SIMG) and were monitored via video in a daycare setting. The drop heights varied between 0.01 and 1 m. No one was critically wounded in the fall, but one child did have minor injuries. Kakara et al. [9] outfitted a licensed creche facility with an embedded video surveillance system and equipped test subjects with accelerometer gyroscope sensors. The head injury criterion (HIC) was calculated using fall simulations via FEM. The result is the biomechanical simulation cannot be utilized to estimate the severity of fall-related injuries without actual fall data. Most of the sensor-based falls took place in a “safe” environment, and the researcher safeguarded children at a predefined rate for ethical reasons, resulting in minimal injury events required for experimental accuracy. There is a need to enhance the precision of the experiment.

Utilizing wearable sensors, measuring the mechanical indicators of a toddler’s head is conducted. This process involves discretizing the solution area into a grouping system of units recombined in a computer to generate a finite element model [10,11]. Angela et al. [12] investigated the effects of altering fall environment and child proxy characteristics on fall kinetics and probable injury consequences using a bed fall FEM. Environmental measures (bed height, onset force, and impact surface stiffness) are more sensitive to changes in injury outcome indicators than alternative parameters (body segment stiffness, neck stiffness, and head stiffness). Fahlstedt et al. [13] used FEM to test the effects of three playground surface stiffnesses and three head hit locations on seven age groups ranging from 1.5 to 18. These techniques need to learn about children’s injury tolerance and biomechanical responses, which limits their fidelity. Their models have stiffer heads and necks than real children but share anthropometric characteristics with a 12-month-old youngster [10,11,12]. Computer models are discrete representations of actual occurrences, so it should be stressed. Hence, this class of models may need more accuracy regarding bed falls.

Children are hesitant to wear sensors daily due to discomfort and limited mobility, and these expensive devices are often subject to complex home environments. Therefore, vision-based fall detection approaches have been popular in recent years. Skeletal information [14,15] was retrieved from RGB photos using a deep-learning model [16,17,18] like OpenPose. A recurrent neural network model, such as a Region Convolutional Neural Network (R-CNN), was used to learn the movement features [19]. Spatio-Temporal Graph Convolutional Networks (STGCN) [20], and a one-dimensional convolutional neural network mode (1D-CNN) [21]. This class of techniques offers high detection precision and recall. Still, their processing speed is insufficient for real-time detection and fall injury assessments of children, which requires a predictive analysis of changes over time. Time series-based models have high precision and speed advantages, among which LSTM [22,23,24] obtains multimodal feature fusion for fall detection from skeleton frame sequences, which satisfies the need for fall injury assessment. Lin et al. [24] reported that the accuracy of the Recurrent Neural Network (RNN), LSTM, and Gate Recurrent Unit (GRU) models did not improve after interpolation, indicating that the interpolation procedure does not have the desired effect when key point data are lacking.

**Figure 1:**
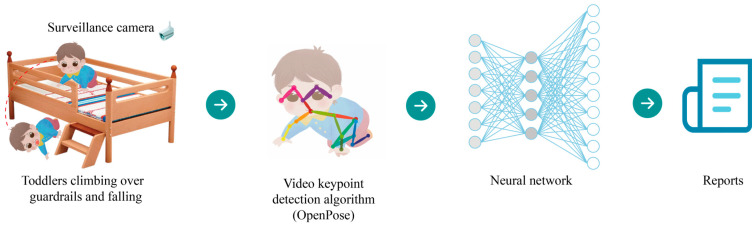
General structure of child head injury assessment system.

In light of the preceding discussion, to make intelligent child fall injury assessment more accessible and practical. We devised an algorithm via real-time monitoring to estimate the impact force on a falling child’s head. As depicted in Figure 1, we can assess whether or not they sustain injuries and issue a real-time alarm warning.

For the self-built dataset, we selected 200 RGB-captured daily videos of 13- to 30-month-old toddlers playing near furniture edges or guardrails and upside-down falling. These recordings ranged in length from 10 min to 15 s. One hundred parents consented to use video surveillance content for research purposes. Five hundred video clips can be cut out based on the rapid head movement of these videos. The dataset’s diversity was enhanced, where the children’s beds’ lighting conditions, camera settings, and furnishings varied. It may improve the framework’s generalization capacity. The processed falling video data is fed into a 3D transform model to identify the features. The recognition results are output via the regression layer to construct a model for assessing the seriousness of the head injury based on Head Impact Criteria (HIC) [25]. Subsequently, the trained classification model is employed to carry out the assessment. Finally, the dataset from another 300 video clips of children’s falls is utilized to test the performance of the framework.

The following are the main contributions of this research:

1. A two-stage deep-learning-based architecture for high-precision detection is proposed. The LSTM neural network and 3D transform model can correct the coordinates of the omitted 2D skeleton points and derive the 3D stereo coordinates by combining the spatial and temporal information of the key points. We hope to decrease missed or inaccurate detections brought on by complicated environments, small target sizes, and density distributions. It seeks to bring intelligent child fall in-injury assessments one step closer to being convenient and speedy.
2. A HIC-based approach for classifying damage degree is developed based on the triaxial acceleration of the human head center drop.
3. A dataset of 500 video clips of children’s falls is generated using 200 real-time daily videos, and another test set of 300 video clips is used to evaluate the performance of the system. These videos encompass a range of lighting conditions, camera settings, and interior furnishings. It levels the dataset’s diversity and boosts the framework’s generalization ability.
4. The feasibility of a real-time AI technique for assessing head injuries in falls is proven. After future upgrades and optimizations, it is implementable on hardware platforms such as intelligent surveillance cameras.

The rest of this essay is structured as follows. The approach is described in Section 2. Experiments are performed, and the outcomes are discussed in Section 3. Section 4 contrasts the methodology and accuracy of our classification system to those of fall injury classification systems. Section 5 outlines the conclusions of this work and possible further research.

## 2. The Proposed Method

We attempted to extract the key point parameters before designing the framework. The compared algorithms were YOLO and OpenPose, and YOLO failed to attain the expected results, as evidenced by the following factors: while capturing the target child, other interfering objects are also captured, impeding the algorithm’s ability to detect children; the algorithm cannot capture the child if there is excessive feature occlusion or low light intensity. These issues necessitate the creation of YOLO plugins. Therefore, OpenPose was ultimately chosen to extract the parameters of the child’s objective.

The framework is divided into two stages: extraction of kid key point information and classification of brain injury evaluation.

The process commences with selecting and cutting fragment frames depicting the child’s fall. These frames are subsequently sent into OpenPose and merged with LSTM to identify the child’s key point information within the input image. Then, the frames are passed via the 3D transform model to estimate the 3D human pose. Ultimately, the target transformation results are integrated with the previous stage’s output key point information to complete the 3D skeletal point. It implies that the estimation of the head point positions can be deduced based on the body joints, even if the child’s head is concealed.

The head acceleration is computed using the head point’s coordinates, then entered into the HIC value calculation method, and the degree of head injury is determined by comparing it to the international standard value. Brain injury severity can be used to designate the human body as safe or dangerous.

**Figure 2:**
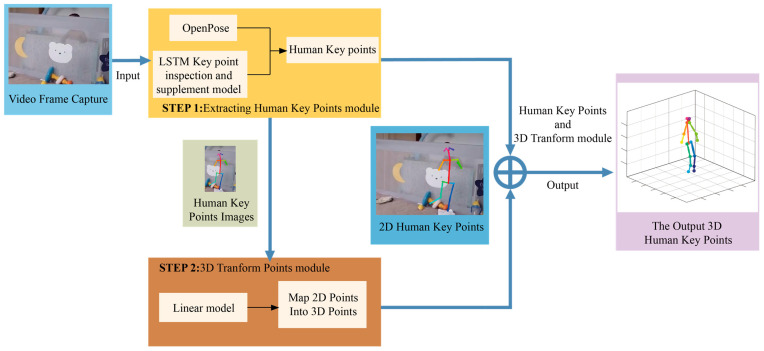
General structure of assessment system using OpenPose and LSTM.

Using these two elements, we create an algorithm with temporal continuity and an exceptional classifier for identifying potential threats. Figure 2 shows the pipeline of the framework.

### 2.1. Open Pose Obtains Human Body Skeleton Information

Open Pose can demonstrate stable tracking in the presence of underbody occlusion or non-frontal lobe tracking to extract human key points from datasets [26]. It can extract 25 human key points from 2D pictures in low-light conditions, as shown in Figure 2. It overcomes the detection distance limitation compared to the Kinect sensor-based method for obtaining fall recognition points.

### 2.2. 2D Key Points Inspection and Repair

Due to the fact that the coordinates of 2D key points recognized via Open Pose are readily obscured by other objects or lighting concerns in the home, the coordinate points may be disregarded or misled, affecting subsequent 3D coordinate estimations; therefore, the 2D joint points must be verified and supplemented.

Only spatial information variables are considered in Open Pose’s 2D key point extraction process. We integrate each frame’s spatial and temporal information to ensure no critical key points are neglected. We use the LSTM [27] to learn long-term dependencies between data based on the correlation of human joint coordinates in continuous time increments and extract complete 2D key point coordinates frame by frame.

This study mathematically describes its forward propagation:

(1) $$F_{t}=\sigma \left(T_{t}\times W_{f}+h_{t-1}\times {\left(Wh\right)}_{f}\right)$$

$T_{t}$ is the input forgetting gate at time *t*, ${\;W}_{f}$ is the weight corresponding to the input, $h_{t-1}$ is the hidden state at time *t*−1, ${\left(Wh\right)}_{f}$ is the matrix of weights within the hidden state, and σ is the function that makes the final result at that gate.

(2) $$I_{t}=\sigma \left(T_{t}\times W_{t}+h_{t-1}\times {\left(Wh\right)}_{i}\right)$$

(3) $${\tilde{C}}_{t}=\tanh\left(T_{t}\times W_{c}+h_{t-1}\times {\left(Wh\right)}_{c}\right)$$

$T_{t}$ is the input at time *t*,${\;W}_{i}$ is the weight corresponding to the input, $h_{t-1}$ is the hidden state at time *t*−1, ${\left(Wh\right)}_{i}$ is the matrix of weights within the hidden state, and σ is the function that gives 0 or 1 as the final result at that gate.

(4) $$C_{t}=F_{t}\times C_{t-1}\times I_{t}\times {\tilde{C}}_{t}$$

(5) $$O_{t}=\sigma \left(T_{t}\times W_{o}+h_{t-1}\times {\left(Wh\right)}_{o}\right)$$

(6) $$H_{t}=O_{t}\times \tanh\left(C_{t}\right)$$

(7) $$\left\{\begin{array}{l} C_{t-1}\times F_{t}=0,F_{t}=0\\ C_{t-1}\times F_{t}=1,F_{t}=1 \end{array}\right.$$

where $f_{t}$ represents the forget gate, $i_{t}$ is the input gate, and ${\tilde{C}}_{T}$ denotes the candidate state. The forgetting gate decides whether or not to keep the information from the previous timestamp based on Equation (1). The input gate employs Equation (2) to compute the significance of the information and utilizes Equation (3) to incorporate the updated information. Equation (4) can also be employed to modify the state of a cell. Ultimately, the output gate utilizes Equation (5) to generate the output and simultaneously changes the existing hidden state by Equation (6). The input sent to the LSTM unit is denoted as *F*. This input represents the key point data about the missed detection occurring in the kth frame of the video. As demonstrated in Equation (7), input values with a value of 0 for the variable ${\;F}_{t}$ = 0 are disregarded, whereas input data with a value of 1 for ${\;F}_{t}$ = 1 are preserved. The LSTM network was configured to memorize up to 100 dependencies, which proved adequate for achieving the desired results. The results were then forwarded to the fully connected layer, analyzing the nonlinear relationships between the higher-level features.

Algorithm 1 details the 2D key point inspection and repair process.
**Algorithm 1:** Correction of Skeleton Key Point Coordinates**Input:** Bone point coordinate matrix’*F*’, size’$T\times N_{t}\times 2’$ **Output:**
$\mathrm{Missing}\;\mathrm{point}\;N_{t}\times 2$ **Procedure:** 1: **Input:** Enter key point data’*F*’; 2: **Forget gate:**
$F_{t}$ is 0 or 1 **If** $C_{t-1}\times F_{t}$ = 0, The input value is reserved **Else:** The input value is forgotten; 3: **Input gate:** By the sigmoid and tanh functions, determines the value affecting $C_{t}$; 4: **Output gate:** By the sigmoid function, output $C_{t}^{\prime }$, then $C_{t}^{\prime }\times O_{t}$; 5: Combine T results for linear regression and out the missing point’s coordinates.

### 2.3. Map 2D Key Joint to 3D Positions

**Figure 3:**
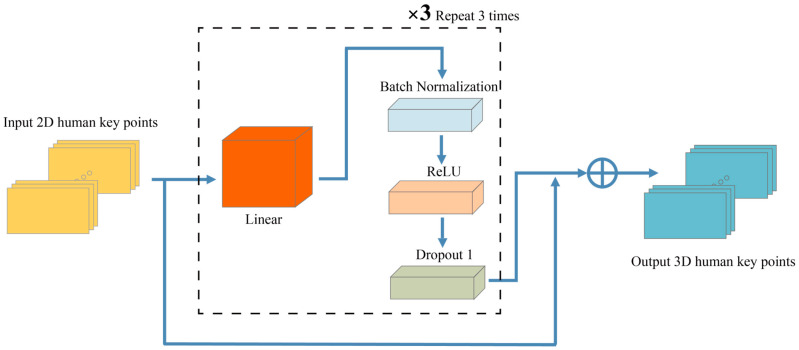
Map 2D points into 3D points.

After obtaining the 2D key point coordinates, we wish to use the 2D input to estimate the human key point locations in 3D space. The method conveys less information in 2D detection, but its low dimensionality reduces overall training time and greatly accelerates network design and the search for training hyperparameters. The value of the *z* coordinate is derived from the known 2D coordinates of the key point (*x, y*), as shown in Figure 3.

Our method is founded on a simple, deep, multilayer neural network. The network consists of a linear layer, serial normalization, rectified linear units (RELUs), and dropout. It is performed thrice, with the last link connecting the two sections [28]. Our method accepts a 2D matrix of joint positions as input and generates a series of 3D joint positions. The objective is to identify a function that minimizes prediction error across an *N*-pose set:

(8) $$F^{*}=\arg\underset{h}{min}\frac{1}{N}\sum_{i=1}^{N}L\left(h\left(x_{i}\right)-y_{i}\right)$$

Input is a sequence of 2D points $x\in R^{2n}$, while output is a sequence of 3D points $y\in R^{3n}$. $x_{i}$ are the 2D point coordinates generated via OpenPose during camera image processing.

Batch normalization and dropout are utilized to enhance the efficacy of our system, but this results in a slight increase in training and testing time. Additionally, in conjunction with batch normalization, we set a restriction on the weights of each layer so that the maximum number of paradigms is fewer than or equal to 1. The training initiates a 0.001 percent learning rate and exponential decay with 32-unit batches.

The coordinate conversion is shown clearly in Algorithm 2.
**Algorithm 2:** Convert 2D to 3D coordinates**Input:** 2D bone point coordinates **Output:**
$Z_{i}$ 1: Enter the linear layer and increase its dimension by 1024; 2: Standardize for batch processing and discard; 3: Enter ReLUs for activation processing; 4: Select the Z value with the minor error from it; 5: Entering the linear layer once more, generating an output of size 3 n; 6: Combine with input and output the result.

### 2.4. Values Calculated for Head Injuries

Through research, a wealth of knowledge on brain injury biomechanics has been accumulated, and different injury evaluation standards have been proposed for various types of head injuries. The renowned Wayne State Tolerance Curve (WSTC) [29] has become the basis for most acknowledged craniocerebral tolerance indices. It explains the connection between linear head acceleration, acceleration duration, and the onset of concussion. Gadd (1966) later proposed the severity index (SI) [30] based on the WSTC, and Versace made alterations to the SI as a head injury criterion (HIC) in 1971. It is the most frequently used injury criterion and is calculated as a function of the duration of acceleration at the head’s center of gravity [31]. In 2011, Hideyuki proposed the Rotational Injury Criterion (RIC) [32], derived from HIC by substituting resultant angular acceleration for resulting linear acceleration. However, since this paper is based on visual detection from a single surveillance camera in daily life, the viewpoint and number of equipment are insufficient to obtain accurate angular acceleration. Consequently, this paper employs HIC as the evaluation method for children’s fall injuries.

From the head acceleration on the center of mass, the following formula can be used to calculate the HIC.

(9) $$HIC=\left(t_{2}-t_{1}\right){\left[\frac{1}{t_{2}-t_{1}}\int_{t_{1}}^{t_{2}}a\left(t\right)dt\right]}_{max}^{2.5}$$

where $t_{1}$ and $t_{2}$ are the initial and final impact times (in seconds), and *a (t)* is the head acceleration at the point of impact (in g/s, with g being the standard gravitational acceleration). $t_{2}-t_{1}$ is the impact time, which is restricted to 36 ms or 15 ms based on historical data and Federal Motor Vehicle Safety Standard 208 in order to guarantee the maximum HIC. Due to the scarcity of time, we employ a 15 ms time limit.

Table 1 shows that the specific HIC criteria are 250, 700, and 1000, defining a spectrum of injury severity levels [33,34]. We employ the conventional HIC criteria as a classification standard for injuries sustained by toddlers during overturning falls.

Using previously obtained 3D coordinates, we estimated the head’s acceleration. The obtained acceleration data was then used to calculate the HIC by fitting the curve in MATLAB to identify and evaluate the fall-related head injury. As shown in Table 2, it is based on the HIC-derived head injury threshold. There are four categories of injury severity: no injury, minor injury, moderate injury, and severe injury. This value functions as the label for the training data that follows.

### 2.5. Classification Based on Machine Learning

We compare two standard classification algorithms, random forest (RF) and support vector machine (SVM), to determine the most effective method for assessing fall injuries.

SVM provides small sample pattern identification advantages and is initially utilized for binary classification. A kernel function linearizes and categorizes the input feature vector’s characteristics by projecting them onto a high-dimensional space.

Several advantages of the RF algorithm have been highlighted in the literature. RF yields high output precision and is resistant to overfitting [35,36]. It is computationally faster than other algorithms, such as SVM. In addition, it allows us to select the most essential variables [36] and eliminate the least important attributes [35,37].

## 3. Experimental Setup

### 3.1. Dataset and Test

**Figure 4:**
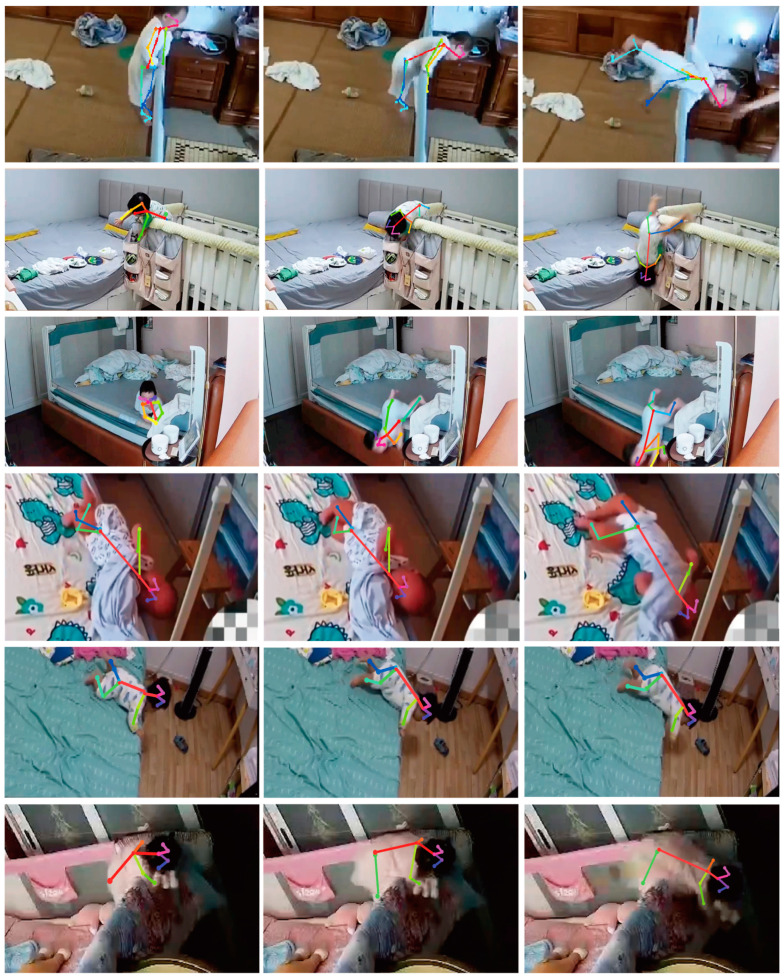
Images in self-build fall dataset of toddlers.

The fall event is employed to assess the effectiveness of the proposed methodology. A total of 200 videos were obtained from parents, capturing the daily activities of toddlers aged 13 to 30 months. These videos specifically focus on instances where the toddlers tumbled from guardrails. The sample comprises 100 toddlers and exhibits diverse lighting situations, camera settings, and interior decor. Including various data in the dataset enhances its diversity and improves the generalization capability of the framework. Several of these are illustrated in Figure 4. Each video lasts 10 min to 15 s. And these depict children’s daily activities, such as falling forward or backward on the bed or being captured after falling more than once in one video. Therefore, we can sample two to three video clips from each video depending on falling forward or backward with various outcomes. A total of 500 video clips of children extracted from 200 videos were divided into an 8:2 ratio into a training and validation set. We prepared an additional set of 300 video clips (test set) of children’s daily falling at home from their parents to evaluate the framework’s performance.

Based on the size of the dataset and the training pace, the batch sizes and Epoch are set to 16 and 300, respectively. The dataset photos are uniformly scaled to 640 × 640 pixels and sent through the network for training the foreground extraction model. The beginning and ultimate learning rates are set at 0.01 and 0.10. The momentum is adjusted to 0.937 to avoid overfitting, and the weight decay coefficient is set to 0.0005 to prevent the model from attaining a local maximum. The toddler’s key point model is trained using images from the dataset scaled to 300 × 300 pixels. The weight decay coefficient and minimum and maximum learning rates are set to 0.0005, 0.0002, and 0.02, respectively.

To evaluate the performance of our proposed model, there are four distinct possibilities within the classification of head injuries.

The first is when the system correctly categorizes damage following a fall.

The second scenario involves an algorithm that wrongly labels a fall event that did not result in injury as an injury.

The third scenario involves a fall incident and an injury not recognized by the system.

In the fourth type, no fall event occurred; hence the algorithm did not divide it.

Accordingly, there are four categories. *TP*, *FP*, *TN*, and *FN*.

True Positive (*TP*): Injury occurred, and the equipment correctly classifies injury.

False Positive (*FP*): No injury happened; however, the equipment misclassified it.

True Negative (*TN*): No injury occurred, and the equipment correctly classified it.

False negative (*FN*): Injury occurs; however, the equipment misclassified it.

The classification’s reliability was evaluated using sensitivity, specificity, accuracy, precision, and F-score to assess these four cases.

(10) $$Sensitivity=\frac{TP}{TP+FN}$$

(11) $$Specificity=\frac{TN}{TN+FP}$$

(12) $$Accuracy=\frac{TP+TN}{TP+TN+FP+FN}$$

(13) $$Precision=\frac{TP}{TP+FP}$$

(14) $$F-Score=\frac{2TP}{2TP+FP+FN}$$

These five metrics are performance indicators in classification selection and model evaluation. Among these is sensitivity, which characterizes the model’s responsiveness to damage classification. Specificity refers to the model’s capacity to prevent misidentification; the more significant the index, the less likely the model will be misidentified for another. These two factors can intuitively highlight the categorization model’s properties. Consequently, they can be used as a more rigorous reference standard.

### 3.2. Experimental Results Analysis

**Figure 5:**
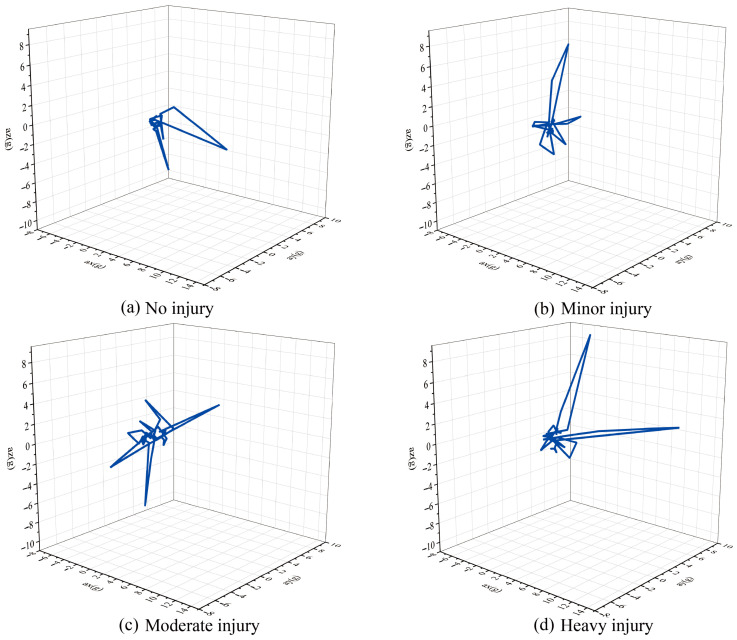
The change of acceleration in x, y, and z directions.

Figure 5 displays the change of acceleration in 3D data from our self-build fall dataset when four types of injury events occur: no injury, minor injury, moderate injury, and heavy injury.

Rapid changes occur in the coordinates of the body during the descent. Consequently, the curve in the figure appears to change swiftly and dramatically. After the skull hits the ground, a secondary collision will occur due to sliding or bouncing. This is also related to the impact surface stiffness [12]. Therefore, the curves at this stage are clustered in the figure to create a cluster.

Figure 5d depicts a scenario in which a head injury is severe. Compared to Figure 5a–c, Figure 5d’s acceleration graph has a more extensive change area in the 3D coordinate system. It represents a more significant acceleration change, possibly related to falling height, toddler weight, and onset force. However, clusters representing secondary bounces in Figure 5d did not differ substantially from the other three cases. In our framework, we cannot infer the association of ground stiffness with injury severity qualitatively and quantitatively. It requires AI identification of ground material hardness through visual detection.

We utilized the additional set of 300 video clips of children’s daily falling to evaluate the model’s dependability. We have chosen three classifiers to compare classification outcomes, including random forest (RF) [37,38,39,40] and two kernel functions in the SVM [41,42,43,44,45,46] classifier: linear and RBF [42,47]. Table 3 outlines the test results.

**Figure 6:**
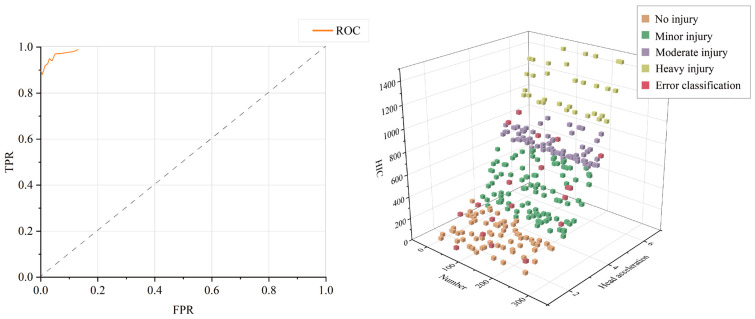
Test results of 300 samples (SVM).

According to Table 3, RBF achieved the lowest classification results and is manifestly inapplicable to the research. Figure 6 depicts the classification results using linear SVM. Therefore, the four categories of No injury, Minor injury, Moderate injury, and Serious injury were renamed as 0, 1, 2, and 3, respectively. Three out of ninety No injury samples were misclassified as Minor injury, while seven out of eighty-four Minor injury samples were misclassified as No injury. Two samples are misclassified as Moderate injuries, while four out of 82 Moderate injuries are misclassified as Minor injuries. Two samples out of 44 with Heavy injury are incorrectly classified as having Moderate injury. While the method obtains a precision of 98.48%, its accuracy is only 93.67%, and the remaining evaluation criteria are unimpressive.

**Figure 7:**
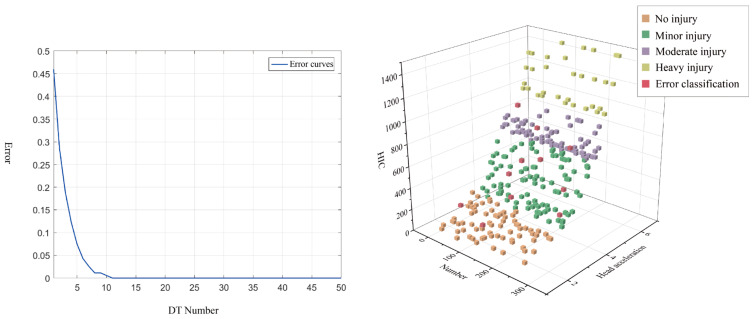
Test results of 300 samples (RF) and Error Curves.

As demonstrated in Table 3, the random forest outperformed other classifiers regarding sensitivity, specificity, accuracy, precision, and F-score; its classification results are depicted in Figure 7. Two out of ninety samples of No injury are misclassified as Minor injury, two out of eighty-four samples of Minor injury are misclassified as No injury, and three out of eighty-two samples of Moderate injury are misclassified as Minor injury. Three of eighty-two samples of Moderate injury are incorrectly categorized as Heavy injury. The Heavy injury of 44 samples is accurately categorized. Figure 7 depicts the visual classification outcomes derived from Random Forest (RF) and Table 3 for the fall injury assessment test conducted on the fall dataset.

This study’s technique effectively identifies human head injuries with a low rate of missed detection, achieving 96.67% accuracy and 99.02% precision; it has a specificity of 97.78% and an F- grade. At the same time, it provides a specificity of 97.78% and an F-score of 97.58%, indicating that this procedure effectively distinguishes between various injuries and generates fewer false positives. The four levels of head injury have been correctly classified by the classification system.

The random forest (RF) demonstrates a high level of efficacy in performing classification tasks, whereas the approach of evaluating fall injuries through the measurement of head acceleration also exhibits effectiveness.

## 4. Discussions

This research proposes a real-time head fall injury assessment based on HIC values. The detection procedure consists of two phases, intending to accurately detect toddler injuries from falling in real-time. Phase I begins with extracting the video frames. The data of joints is obtained by processing video captured by surveillance with Open Pose. The LSTM neural network and 3D transform model are then used to integrate key spots’ frame space and time information. In phase II, the head acceleration is derived and inserted into the HIC value calculation. The experiment results demonstrate that the system has an accuracy of 96.67%, sensitivity is 96.19%, and specificity is 97.78%. The proposed detection system can be extended functionally and structurally to outdoor environments. For example, in community playgrounds, surveillance cameras near slides and climbing frames could monitor the occurrence of head injuries and issue a real-time alarm warning.

Table 4 compares the classification results of the proposed method to those of other classifiers, such as ANN [48,49], CNN [50], and STGCN+1D-CNN [21]. The proposed framework’s classification accuracy on self-built datasets is 96.67%. Similar to this, other classifiers’ classification accuracy using the self-built dataset is 93.14%, 94.3%, 96.43%, and 95.8%, respectively. The classification accuracy of STGCN+1D-CNN [21] with URFD is 96.53%. Thus, the proposed method attains a slight advantage compared with other classifiers.

Even though the offered method produces good detection results, it has shortcomings.

1. As we are using home environment monitoring, some interior furnishings obstruct the camera angles, resulting in insufficient data due to the absence of key joints. We will optimize the ability of our framework to repair small-size toddlers’ skeleton joints.
2. Since our framework needs to be uploaded to the server for processing, we should also perform real-time optimization to increase the model’s inference efficiency. Throughout each iteration, the convergence rate of the optimization techniques should be improved.
3. Our framework does not allow us to deduce how the stiffness of the ground affects the severity of injury. AI ground hardness identification via visual detection and the effect on the harm value is required in the coming work.
4. In practice, it is necessary to determine whether a fall has occurred before deciding the presence of a head injury. So, a vision-based fall detection system is required in the framework in the future.

## 5. Conclusions

The feasibility of a real-time AI technique for assessing head injuries in falls via monitoring is proven. It encourages advancing child injury detection research in the direction of practicability. The detection procedure consists of two phases, key points repair and 3D transform, a classifier of injuries based on HIC. In phase I, the LSTM neural network and 3D transform model integrate key spots’ frame space and time information and repair the coordinates of the missing skeletal point. In phase II, the head acceleration is derived and inserted into the HIC value calculation to establish the classifier. Reliable video surveillance of falls in children’s daily lives is crucial to improving our accuracy. Nearly 14.7% of the falls in our 300 video frames (test set) involving toddlers resulted in serious injuries (requiring medical treatment). This is confirmed after parental verification. Compared with the traditional head injuries assessment method, toddlers’ discomfort with wearable sensors is addressed. It also solves the lack of accuracy in injury detection of the FEM model. The experiment results demonstrate that the system has an accuracy of 96.67%. sensitivity is 96.19%, and specificity is 97.78%. Our solution has a relatively high detection accuracy for toddler fall-related injuries and a substantial application value.

## Figures and Tables

**Table 1:** The HIC values correspond to the injury table.

Head Injury Criterion	Injury Type
250	cerebral concussion
700	Serious injury probability: 5% (Ais = 4)
1000	The probability of developing a malignant skull fracture: 33%

**Table 2:** Classification of head injury severity.

Head Injury Criterion	Injury Type
α < 250	No injury
250 < α < 700	Minor injury
700 < α < 1000	Moderate injury skull fracture: 33%
1000 < α	Heavy injury

**Table 3:** The experimental calculation results.

	***Sensitivity***	***Specificity***	***Accuracy***	***Precision***	***F-Score***
LibSVM	92.38%	96.67%	93.67%	98.48%	95.33%
RBF	91.43%	94.44%	92.33%	97.46%	94.35%
RF	96.19%	97.78%	96.67%	99.02%	97.58%

**Table 4:** Comparison of our suggested algorithm to other methods.

Reference	Theme	Dataset	Approach	Accuracy
Dusenberry et al. [48]	predict head injury	self-built	ANN	93.14%
M Sinha et al. [49]	predict head injury	self-built	ANN	94.3%
Yhdego et al. [50]	fall detection	self-built	Sensor data + CNN	96.43%
Amsaprabhaa et al. [21]	vision-based fall detection	URFD, self-built	STGCN+1D-CNN	96.53%, 95.8%
Ours	head injury assessment	self-built	LSTM+3D transform model	96.67%

## Data Availability

Not applicable.

## Abbreviations

The following abbreviations are used in this manuscript:

RGB	Red, green, blue
RNN	Recurrent Neural Network
LSTM	Long short-term memory
GRU	Gate Recurrent Unit
STGCN	Spatio-Temporal Graph Convolutional Network
CNN	Convolution neural network
FEM	Finite element method
WSM	Wearable sensor-based method
DANN	Deep Adversarial Neural Network
HIC	Head Impact Criteria
SIMG	Sensor-laden headbands
ReLU	Rectified Linear Unit
SVM	Support vector machine
RBF	Radial basis function
RF	Random forest
DT	Decision tree
